# The Matrix of Mitochondrial Imaging: Exploring Spatial Dimensions

**DOI:** 10.3390/biom15020229

**Published:** 2025-02-05

**Authors:** Irene M. G. M. Hemel, Ilja C. W. Arts, Michelle Moerel, Mike Gerards

**Affiliations:** Maastricht Centre for Systems Biology (MaCSBio), Maastricht University, 6229 EN Maastricht, The Netherlands

**Keywords:** imaging, microscopy, mitochondria, MRI

## Abstract

Mitochondria play a crucial role in human biology, affecting cellular processes at the smallest spatial scale as well as those involved in the functionality of the whole system. Imaging is the most important research tool for studying the fundamental role of mitochondria across these diverse spatial scales. A wide array of available imaging techniques have enabled us to visualize mitochondrial structure and behavior, as well as their effect on cells and tissues in a range from micrometers to centimeters. Each of the various imaging techniques that are available offers unique advantages tailored to specific research needs. Selecting an appropriate technique suitable for the scale and application of interest is therefore crucial, but can be challenging due to the large range of possibilities. The aim of this review is two-fold. First, we provide an overview of the available imaging techniques and discuss their strengths and limitations for applications across the sub-mitochondrial, cellular, tissue and organ levels for the imaging of mitochondria. Second, we identify opportunities for novel applications and advancement in the field. We emphasize the importance of integration across scales in mitochondrial imaging studies, particularly to bridge the gap between microscopic and non-invasive techniques. While integrating these diverse scales is challenging, primarily because such multi-scale approaches require expertise that spans different imaging modalities, we argue that integration has the potential to provide groundbreaking insights into mitochondrial biology. By providing a comprehensive overview of imaging techniques, this review paves the way for multi-scale imaging initiatives in mitochondrial research.

## 1. Introduction

Since the discovery of mitochondria in 1857 [1], a wide variety of techniques have been used to study their structure and functioning. Understanding the structure and dynamic behavior of mitochondria is pivotal in order to gain insights into the wide range of physiological and pathological processes in which mitochondria are involved. Imaging has been widely used as a tool to study mitochondria, offering insights into their morphology, structure, functioning and behavior in vitro and in vivo. For instance, the double membrane and cristae structure were discovered using electron microscopy (EM) [2]. Furthermore, various imaging techniques are used in the clinical diagnosis of mitochondrial diseases [3].

Mitochondrial imaging can be performed on various spatial scales, each with a specific purpose. Sub-mitochondrial imaging is primarily used to visualize cristae structure and protein distribution, while mitochondrial imaging at a cellular level can be used to track morphological changes and mitochondrial functioning [4,5,6]. Tissue sections are commonly used in clinical applications in order to identify mitochondrial deficiencies [7]. Additionally, imaging of mitochondria in tissues is useful for the mapping of reactive oxygen species (ROS) and mitochondrial calcium, informative of mitochondrial health and functioning. Similarly, in vivo imaging of organs is regularly applied for diagnostic purposes [3], but can also be used to track treatment effectiveness. It is furthermore used to track changes in mitochondrial membrane potential, and to study the effect of mitochondrial dysfunction on organ structures [8,9].

A diverse array of techniques is available for mitochondrial imaging, including EM, confocal microscopy, light-sheet microscopy and MRI. Each technique offers unique advantages tailored to specific research needs. While it is important to select a technique suitable for the scale and application of interest, this is not straightforward due to the vast amount of techniques available. Additionally, while some techniques are readily available and easy to operate, others require specific expertise.

Aside from selecting a suitable technique for a specific spatial scale, integration of multiple spatial scales within one study has the potential to provide more comprehensive insights into mitochondrial biology. For instance, combining imaging at a cellular and organ level can provide insights into the changes in mitochondrial morphology that underlie the changes observable in the brains of patients with mitochondrial diseases. Currently, the application of multi-scale approaches is very limited, and almost exclusively occurs between cellular- and tissue-level imaging. Most likely, this due to the fact that the expertise of an individual researcher or research group is often restricted to one or a few imaging techniques. This is the case for the various microscopy types applied for sub-mitochondrial-, cellular- and tissue-level imaging. The gap in expertise becomes significantly larger between microscopic and non-invasive imaging techniques applied at the organ level. This review describes mitochondrial imaging techniques across spatial scales, and highlights opportunities for mitochondrial imaging within and across these scales. By providing an overview of the available and most suitable imaging techniques for each application, future multi-scale endeavors in mitochondrial imaging may be facilitated.

## 2. Imaging Sub-Mitochondrial Structures

**Figure 1:**
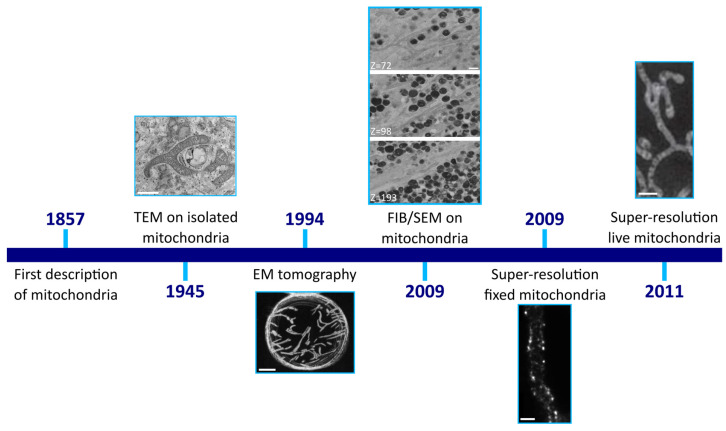
Sub-mitochondrial structure visualization. This timeline provides a historical overview of the various sub-mitochondrial imaging techniques. First FIB/SEM paper on mitochondria [17]. EM tomography image from Mannella, C.A. [18], super-resolution fixed mitochondria from Schmidt, R. [19] and super-resolution live mitochondria from Shao, L. [20], all obtained with permission. TEM = transmission electron microscopy; FIB/SEM = focused ion beam/scanning electron microscopy. Scale bars from left to right equal 1 µm, 250 nm, 100 nm, 250 nm and 1 µm.

Imaging techniques have been used to study mitochondria for more than a hundred years. Initially, the observation of cellular structures was only possible with light microscopy. This resulted in the first observation of small structures in muscle cells, which would later be called mitochondria. While the technology at that point did not yet allow for a visualization of sub-mitochondrial structures, these early studies showed that the size and shape of mitochondria varied from short granule-like structures to rods and small filaments, depending on the cell type [10]. The invention of electron microscopy made it possible to observe the mitochondrial structure in more detail. In early investigations, transmission electron microscopy (TEM) was performed on isolated mitochondria, revealing small fragments present inside some mitochondria (Figure 1) [10]. However, the precise structure of these sub-mitochondrial fragments remained unclear due to the lack of sectioning in the previous research. The first descriptions of cristae and the mitochondrial matrix were provided after the application of TEM on sectioned mitochondria across various cell types [2]. Since then, the quality and resolution of TEM images has increased drastically, reaching spatial resolutions from 300 pm in the 1970s to <50 pm today [11]. Current TEM technologies even allow magnifications of 50 million times [12], enabling the detailed assessment of mitochondrial cristae structure. This technique continues to play a role in current mitochondrial research, particularly in examining the differences in mitochondrial structures of fixed tissues and cells and in localizing sub-mitochondrial structures and proteins [4,13,14,15,16].

Unfortunately, the application of TEM is confined to very thin sections, typically <100 nm [12], limiting its ability to provide a complete image of the mitochondria and mitochondrial network in a cell. EM tomography represents a partial solution, as it enables the 3D reconstruction of a very small fragment (<5 µm) with a high 3D resolution (1 nm), allowing the visualization of individual mitochondria in more detail than possible with 2D sections (Figure 1). This is achieved through the acquisition of a series of images at diverse tilted angles, which are subsequently reconstructed [21]. This technique was first used in 1994 to visualize rat mitochondria, and presented a 3D reconstruction of the inner mitochondrial structure [18]. Subsequently, EM tomography was applied to further describe the cristae structure, leading to the discovery of cristae junctions [22]. The possibility of a high-resolution 3D reconstruction makes EM tomography especially useful for the detailed reconstruction of organelle and sub-organelle structures [21].

Focused ion beam scanning electron microscopy (FIB-SEM) is an alternative EM-based method that also enables the 3D reconstruction of samples (Figure 1). In contrast to EM tomography, FIB-SEM offers a field of view that is up to 20 times larger, albeit with a distinctly lower 3D resolution (20 nm) [21]. Additionally, since the technique uses scanning electron microscopy (SEM) the maximum possible magnification is limited to 2 million times, while it is up to 50 million times for EM tomography [23]. In FIB-SEM, a slice of the sample is imaged and subsequently removed with the ion beam before the imaging of the next slice. While this process is destructive in nature and time-intensive compared to TEM tomography, it allows for the 3D reconstruction of considerably thicker samples [21]. This makes FIB-SEM suitable for imaging (parts of) a cell and small tissues with a relatively high resolution, allowing the observation of mitochondria in relation to other cellular compartments [21]. Additionally, FIB-SEM can be used for the assessment and quantification of mitochondrial morphology and to study mitochondrial protein localizations [24,25,26].

These various EM techniques allow for the high-resolution imaging of the sub-mitochondrial structures. However, a major downside of EM is that it is restricted to fixed samples, meaning that studying the dynamic nature of mitochondrial processes is impossible with this approach. In contrast, light microscopy can be applied to living cells, but conventional light microscopy cannot reach the required resolution for sub-mitochondrial imaging. The recent development in super-resolution microscopy techniques has opened up this possibility (Figure 1). Currently, there are various super-resolution microscopy techniques available. As structured illumination microscopy (SIM), stochastic optical reconstruction microscopy (STORM), and stimulated emission depletion microscopy (STED) are most commonly used for mitochondrial imaging, these will be discussed in more detail.

SIM has a resolution of around 100 nm, representing a significant enhancement compared to the ~200 nm attainable through conventional light microscopy [27]. SIM is relatively easy to use and can be used for various standard fluorophores without the need for specialized sample preparation [28]. However, because it requires acquiring multiple images with a high signal-to-noise ratio to enable a high-resolution reconstruction, it can result in significant photodamage, ultimately limiting its applicability in live cell imaging [28]. Nevertheless, SIM is still considered the least phototoxic super-resolution microscopy technique [29,30]. Moreover, while the sample thickness is limited to ~5–15 µm compared to the ~50 µm possible with confocal-based techniques, SIM allows faster image acquisition than most forms of laser scanning confocal-based microscopy, resulting in a temporal resolution at the millisecond scale [28,31,32]. For mitochondrial research, SIM has been used in both fixed and living cells. The achievable resolution enables the visualization of inner mitochondrial membrane and cristae dynamics, as well as the observation of groups of cristae and of the interplay between different mitochondrial compartments during mitochondrial dynamic processes [33,34,35]. SIM has also been used to determine the role of micro proteins in cristae architecture and ER–mitochondrial contact sites [4]. Individual cristae, however, are only occasionally observable, depending on the size of the mitochondria and the proximity of cristae to each other [34].

STED and STORM have an even higher spatial resolution, allowing for the observation of individual cristae [34]. Since STED is a laser scanning confocal system, it is particularly useful for thicker samples (>15 µm). However, the imaging speed is substantially lower than that of SIM, as the image is scanned pixel by pixel. This leads to a lower temporal resolution (~1 s) and higher levels of phototoxicity [29,31]. The extent of the increased phototoxicity depends on the specific properties of the lasers in the system, among other factors [29,36]. Within mitochondrial research, STED microscopy has been used to visualize protein distributions across the different membranes and compartments, as was performed for the TOM complexes, VDAC isoforms, and Perilipin 5 [37,38,39]. Furthermore, it allows for the visualization of cristae structure in minute detail, facilitating the monitoring of cristae dynamics during fission and fusion events [36,40]. Additionally, STED microscopy can be applied to visualize the interactions between mitochondria and other cellular compartments, such as the endoplasmic reticulum (ER) or the cytoskeleton, at a high resolution [41].

The third super-resolution technique commonly used in mitochondrial research is STORM. STORM, which boasts an even higher spatial resolution than STED, offering a lateral resolution of 30 nm in living cells and of up to 10 nm in fixed cells, surpassing the 40 nm achievable with STED microscopy for biological samples [42]. A downside is that its image acquisition time is very lengthy, and extensive post-processing is required to reconstruct the final image [42]. While STORM is applicable in living cells, its slow acquisition is suboptimal for imaging mitochondrial dynamics events. For instance, fission events can be captured, but the cristae are less distinguished than with STED microscopy [43]. This is likely due to the constant movement of mitochondria and the reshaping of cristae, which can take place during image acquisition. Nevertheless, the advantage of requiring low light levels renders STORM more suitable for live cell imaging applications where a lower temporal resolution is acceptable [42]. STORM can be used for the assessment of the distribution of mitochondrial proteins and transcripts and can be used in combination with SIM [4,44,45].

The optimal imaging technique for a specific purpose depends on the required spatial and temporal resolution. When a dynamic event is not the interest of a study, the massive increase in resolution with EM is certainly beneficial. The resolution achievable with TEM is not feasible with any other technique, making it the most suitable to visualize detailed sub-mitochondrial structures and interactions with other organelles (Table 1). SEM-based techniques are advantageous when a larger field of view or an increased imaging depth is of interest. Additionally, SEM imaging is faster than TEM and requires less sample preparation, but the magnification and resolution are lower (Table 1). When studying dynamic events, including cristae dynamics, super-resolution microscopy should be used instead of EM, since it allows live cell imaging. SIM is most suitable for live cell imaging, due to its high temporal resolution and relatively low phototoxicity (Table 1). This technique cannot, however, be used for the visualization of single cristae. Although STED microscopy would be most suited for single-cristae visualization in living cells, the high levels of phototoxicity should be taken into account when analyzing the images (Table 1). The effects of phototoxicity on mitochondrial structure are a challenge in sub-mitochondrial imaging in living cells. However, measures can be taken to decrease these effects, such as supplementation of the culture medium with additional antioxidants [29]. Another limitation in the imaging of sub-mitochondrial dynamic events is that the maximum resolution possible with super-resolution microscopy is still distinctly lower than the resolution achievable with EM. This can be partially overcome by using a technique referred to as correlative light and electron microscopy. Using this technique, the event of interest can be observed with fluorescent microscopy and imaged with EM after fixation and further processing. With current techniques, it has even become possible to use correlative light and electron microscopy with serial-section EM tomography, allowing a reconstruction of 3D models in both light microscopy and EM. However, correlative light and electron microscopy is a complex technique that requires spatial information on the event of interest. Due to this complexity, its use is uncommon. Furthermore, precaution in sample preparation is necessary since standard fluorescent microscopy and EM preparation are often mutually exclusive [46].

## 3. Imaging Mitochondria in Cells

Mitochondrial imaging at the cellular level, in contrast to sub-mitochondrial imaging, where the primary aim is to visualize the cristae structure, serves a broader range of applications. Common to the various techniques in cellular mitochondrial imaging is the use of diffraction-limited fluorescent microscopy. That is, all techniques combine light microscopy with fluorescence emitting compounds. Each technique has unique advantages and disadvantages. The most commonly used microscopy types are widefield and confocal microscopy. Widefield fluorescent microscopy is the most basic fluorescent microscopy type. With this technique, the entire sample is exposed to light of a specific wavelength. This results in excitation of the fluorescent labels in a sample, which are then captured with a camera [47]. While this approach allows for fast imaging, the sample thickness is limited to ~15 µm [48] (Table 2). Post-processing and deconvolution can increase the signal-to-noise ratio and improve image resolution [47]. Confocal-based microscopy, on the other hand, makes use of a pinhole to eliminate out-of-focus light, thereby sharpening the image by reducing background noise. Due to this, confocal microscopy allows for sample thicknesses up to ~80 µm, which facilitates the generation of 3D images [31,49]. Of the confocal based techniques, laser scanning confocal microscopy (LSCM) and spinning disk confocal microscopy (SDCM) are the most common. LSCM creates an image by scanning the field of view pixel by pixel, creating high-resolution images. However, this process is slow, especially when multiple iterations are performed to increase image resolution, resulting in increased phototoxicity [31] (Table 2). Additionally, imaging dynamic organelles, like mitochondria, is more difficult since they can move during the imaging process. SDCM addresses this drawback by making use of spinning disks with multiple pinholes, enabling simultaneous imaging at multiple-field-of-view sites. This increases the temporal resolution from seconds to the millisecond scale and decreases phototoxicity. This makes SDCM more suitable for live cell imaging than LSCM, especially when imaging mitochondrial dynamics. However, the increased temporal resolution comes at the cost of a sample thickness limited to 40–50 µm [31] (Table 2). Nevertheless, an imaging depth of 40 µm is more than sufficient to visualize a cellular monolayer.

Other types of microscopy less commonly used on a cell level are two-photon microscopy and light-sheet microscopy. Two-photon microscopy is particularly advantageous in 3D imaging because it only excites photons within the focal plane [49]. This confines imaging-induced sample damage to the focal plane, decreases phototoxicity in thick samples and allows for extended imaging, increasing the sample thickness that can be imaged [49] (Table 2). On the other hand, light-sheet microscopy permits lower illumination intensities and provides an improved signal to noise ratio [50]. This results in minimal sample exposure, enabling fast imaging over long periods of time with decreased phototoxicity [50] (Table 2). A drawback of light-sheet microscopy is that samples need to be transparent to make imaging possible, which requires extra sample preparation for samples that are not naturally transparent. Both two-photon and light-sheet microscopy have been used to image mitochondria in cells [51,52,53]. However, two-photon and light-sheet microscopes are more expensive than the previously mentioned techniques and are not commonly available, restraining their use in mitochondrial imaging. Additionally, the gain in imaging depth with these techniques is not required for most cellular mitochondrial imaging applications, resulting in their limited use at this spatial scale.

Mitochondrial imaging at a cellular level has a wide variety of applications, such as the assessment of mitochondrial morphology, dynamics, and health, as well as the determination of calcium flux and protein localization. In general, each of the aforementioned microscopy types can be applied for all of these applications, but due to their wide availability and relative ease of use, widefield and LSCM are most commonly applied in cellular mitochondrial imaging. However, these microscopy types have drawbacks as, previously described, making them less suitable for 3D imaging and live cell imaging. While the broad suitability of fluorescent microscopy types across applications is useful for researchers, it is also important to be aware of the limitations when selecting a microscopy type for a specific cellular imaging application. In the following sections, commonly used mitochondrial imaging applications and the most suitable types of microscopy will be discussed in more detail.

### 3.1. Mitochondrial Morphology

**Figure 2:**
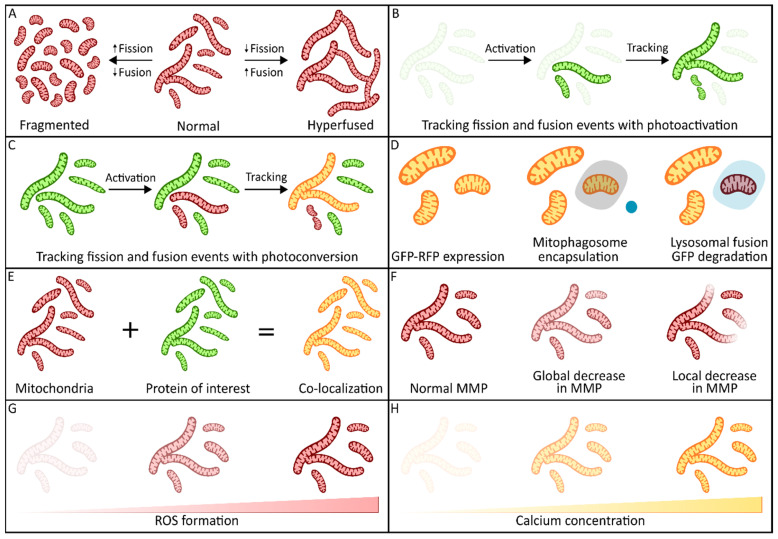
Imaging applications at a cellular level. (**A**) Changes in mitochondrial network morphology as the result of an altered balance between fission and fusion can be determined using imaging and quantification of the mitochondrial morphology. (**B**) Tracking of fission and fusion events using photoactivatable fluorescent proteins. The presence of a signal in non-activated mitochondria is indicative of fusion, while a break in the activated signal indicates fission. (**C**) Tracking of fission and fusion events using photoconvertable proteins. The presence of both activated and non-activated signals indicates fusion events, while the breaking of an activated signal indicates fission. (**D**) Tracking of mitophagy through the loss of green fluorescence upon mitophagosome and lysosome fusion. (**E**) Co-localization of proteins with mitochondria can determined through staining the mitochondria and protein of interest. The overlap between the two stains is indicative of the mitochondrial localization of a protein. (**F**) Mitochondrial membrane potential changes can be visualized through the use of membrane potential-dependent dyes, like TMRM. Both a global change and local decrease in membrane potential can be visualized using imaging. (**G**) Increases in ROS formation are observable through the binding of ROS to fluorescent dyes, which increases the emitted fluorescence. (**H**) Increases in mitochondrial calcium are observable through the binding of calcium with fluorescent dyes, which increases the emitted fluorescence.

Imaging mitochondrial morphology is a key application in mitochondrial dynamics research, especially in monitoring changes under various conditions [5,54,55,56]. While morphology can be imaged in fixed cells using immunofluorescent staining, the fixation of the sample can affect the mitochondrial morphology [57]. Therefore, live cell imaging is preferential in mitochondrial morphological studies. Live cell imaging can be performed in cells stained with specific dyes, or in stable cell lines expressing fluorescent mitochondrial proteins (Figure 2A). Many different live cell imaging dyes are available for this application, including various MitoTracker variants, MitoView, BioTracker, JC-1 and TMRM [58,59,60,61,62]. The uptake of some of these dyes, like TMRM and JC-1, is dependent on the mitochondrial membrane potential [63,64,65]. Consequently, these dyes cannot reliably be used for morphology assessment when an effect on mitochondrial membrane potential (MMP) is expected in a study. Additionally, dyes dependent on MMP can be pumped out of the mitochondria as a result of MMP fluctuations, which can lead to more background signal. Other dyes, such as MitoTracker Red and Green FM, covalently bind to the mitochondria, preventing leakage to the cytosol and leading to less background signal [66]. A downside of these dyes is that the stain fades over time, which makes it very difficult to track mitochondrial morphology changes over time without re-staining. Given this limitation, stable cell lines expressing fluorescently tagged proteins might be a better option, since the signal remains constant in the mitochondria in this case. These cell lines also allow for the targeting of the fluorescent protein to a specific mitochondrial compartment, enabling the visualization of specific membranes—which is not possible with mitochondrial dyes due to the uncertainty of their mitochondrial localization. Nevertheless, stable cell lines expressing fluorescent mitochondrial proteins have drawbacks as well. For instance, the presence of large fluorescent proteins or proteins with ROS scavenging properties, like (e)GFP, can influence mitochondrial behavior and morphology [67,68]. This is especially the case when the fluorescent protein is linked to a full-length protein, while the likelihood of interference decreases when only the mitochondrial target sequence is used.

Regardless of the use of a mitochondrial dye or a stable cell line, the microscopy types suitable for mitochondrial morphology assessment remain the same. In case of 2D imaging, LSCM is preferable over widefield microscopy because of its higher resolution and increased signal-to-noise ratio. However, imaging entire cells in 3D would take several minutes with LSCM. This makes it inconvenient to image a sufficient amount of cells for analysis. Additionally, lengthy imaging results in high levels of phototoxicity, which can alter the morphology under investigation. Additionally, due to the dynamic nature of mitochondria, the morphology can change during the imaging process. Therefore, the use of SDCM is preferable to LSCM for the 3D assessment of mitochondrial morphology. The imaging depth of 2–6 µm required to cover an individual cell [69] can easily be achieved with this technique, and the imaging speed is sufficient to capture an entire cell with limited morphological changes and phototoxicity. Furthermore, 2D SDCM can be used for the continuous tracking of mitochondrial morphology over a short time period (<1 min), and can therefore be used to assess real-time morphological changes or mitochondrial transport. However, when the aim is to track morphology over longer time periods, light-sheet microscopy would be specifically beneficial due to its high imaging speed and low phototoxicity levels.

### 3.2. Mitochondrial Dynamics Events

Beyond general mitochondrial morphology, specific imaging techniques can also track various mitochondrial dynamic processes like fission and fusion events. These events can be quantified with photoactivatable or photoconvertable fluorescent proteins [70,71]. For instance, a photoactivatable version of GFP (PAGFP) increases the fluorescence 100-fold when activated [72]. Using a mitochondria-targeted PAGFP, transfer of activated green signal to unlabeled mitochondria is indicative of a fusion event, while a loss of continuous signal indicates fission events [70,73] (Figure 2B). Additionally, a photoactivatable signal can be combined with additional mitochondrial staining, for instance in the case of MitoTracker. This staining can be used to identify mitochondria that do not undergo fusion, since these will not be visible without the transfer of the photoactivated signal [70]. Photoconvertable proteins, like Dendra2, can be used in a similar way [71,74,75]. Such proteins irreversibly change their emission spectrum when excited with a specific wavelength, for instance from a green to a red excitation, and the fusion and fission events can be monitored through the spreading of the changed fluorescent molecules [71,74,75] (Figure 2C). Another approach, fluorescence recovery after photobleaching (FRAP), involves photobleaching a specific cell area and monitoring the recovery as fluorescent molecules from neighboring mitochondria move in [76]. For all these approaches, a fast imaging technique is required in order to closely monitor the changes, meaning that LSCM and two-photon microscopy are not suitable. Although widefield microscopy is fast enough to monitor the changes, the higher resolution offered by SDCM is beneficial for a more detailed analysis.

### 3.3. Mitophagy

In addition to fission and fusion events, mitophagy can also be assessed using fluorescence microscopy. For example, the mitochondria-targeted RFP-GFP fusion protein can monitor the removal of defective mitochondria through their fusion with lysosomes. That is, RFP-GFP shows both red and green fluorescence under normal circumstances, but the green fluorescence is absent after the mitochondria are fused with the lysosomes due to the change in pH (Figure 2D) [77,78,79]. Similar pH-dependent probes that instead target the mitochondrial DNA have been produced, and can be used when live imaging of mtDNA and in combination with mitophagy [80]. Alternatively, mitophagy can be tracked by the assessment of co-localization between mitochondrial probes with lysosomal markers like LysoTracker or lysosomal proteins fused to a fluorescent protein that is resistant to an acidic pH [81,82]. Depending on the exact research question, various types of microscopy can be applied to study mitophagy. Since mitophagy takes several minutes [83], a fast imaging technique is not required to assess the total amount of mitophagy or to monitor the general process, making LSCM highly suitable. Nevertheless, when more detailed tracking is aimed for, LSCM is suboptimal since the resulting phototoxicity could induce more mitophagy. In that case, a less phototoxic microscopy type, like widefield microscopy or SDCM, would be better.

### 3.4. Co-Localization

Mitochondrial imaging at the cellular level is also crucial for co-localization studies in determining the subcellular localization of proteins of interest [84,85,86]. Such co-localization can be determined using both live and fixed cell imaging. In live cells, protein localizations are most commonly assessed using a fluorescently tagged version of the protein of interest, in combination with a mitochondrial marker like MitoTracker (Figure 2E) [58,84]. However, fluorescently tagging the protein of interest can influence protein localization and behavior [67], and overexpression of these tagged proteins can affect mitochondrial and cellular behavior [87,88]. Alternatively, only the mitochondrial target sequence of a protein of interest can be expressed with a fluorescent tag, but this sequence is not always known, and the presence of this sequence does not always guarantee efficient import into the mitochondria [89]. In fixed cells, co-localization is determined using immune-fluorescent staining [90]. For this, antibodies against the protein of interest are used, although their potential for nonspecific binding must be taken into account when interpreting the localization [90]. Another strategy involves tagging the protein of interest with short tags (FLAG, V5, etc.) and using highly specific antibodies against these tags [91]. However, this method also requires the overexpression of the tagged protein, and the resulting image may not be fully representative of the native protein state. Determining the subcellular localization of a protein in both live and fixed cells can be performed well with LSCM, since it is not a fast-changing process. Furthermore, its improved resolution compared with that of widefield microscopy allows for a more precise determination of protein localization.

### 3.5. Mitochondrial Membrane Potential

Imaging can also be used to assess mitochondrial health and functioning. The mitochondrial membrane potential (MMP) is the result of the proton gradient created by mitochondrial electron transfer activity, and can therefore be used as an indicator of mitochondrial functionality [6]. Assessment of the mitochondrial membrane potential is generally performed using dyes like TMRM or JC-1, which depend on this potential to enter the mitochondria (Figure 2F). High-throughput screenings, as well as longitudinal studies into the MMP, have been performed using this approach [63,92,93,94]. There are also, however, some drawbacks of using microscopes to determine MMP through the analysis of signal intensity in images. Since exposure of the fluorophores results in photobleaching, one needs to ensure that the level of exposure before taking the image, as well as the focal plane, is the same for all images. This is difficult to achieve when the proper focus needs to be established first for each image individually. Ideally, a microscope with a brightfield microscopy option would be used to set the focus. However, this is not possible on every microscope and requires experience. Furthermore, dyes dependent on MMP are not stable in the mitochondria, which can cause differences in signal intensity depending on the way the images are taken. Alternatively, flow cytometry or fluorescence-activated cell sorting (FACS) can be used to assess the MMP in a more consistent and independent manner [95,96]. However, this only allows the assessment of populations of cells and not individual cells or subcellular compartments. When the MMP of individual cells is of interest for a study, determining it through imaging is the only option. Importantly, extensive experience is of paramount importance for the imaging in a consistent and objective manner, and imaging should preferably be performed with a microscope that results in low phototoxicity levels, like widefield microscopy or SDCM.

### 3.6. Reactive Oxygen Species

In addition to MMP, various mitochondrial metabolites can be imaged using specific probes. Mitochondrial reactive oxygen species (ROS) are a significant cause of damage inside cells, play a role in various diseases (e.g., neurological disorders, cancer), and have an important role in cellular signaling and homeostasis [97]. The probes available for ROS imaging (Figure 2G) often target a specific ROS type. For example, MitoPY1 and HQPQ-B are probes that respond to the presence of H_2_O_2_, mitoSOX is used to detect mitochondrial superoxide anions (•O_2_^−^), and TFP can be used for the simultaneous monitoring of ATP and H_2_O_2_ [98,99,100,101]. Imaging of mitochondrial ROS production has been used to determine the effect of a peptide on ROS production in cells from patients with a mitochondrial cardiomyopathy [102], and to demonstrate that cellular H_2_O_2_ homeostasis mostly depends on substrate catabolism in the mitochondria [103]. Since imaging can also induce the production of ROS, it is important to consider the type of microscopy used. LSCM is not very suitable, since it results in high phototoxicity levels, while widefield microscopy and SDCM cause less interference with the measurements and are therefore preferable.

### 3.7. Calcium Imaging

Finally, the last application of imaging for the assessment of mitochondrial functioning that we will discuss is the monitoring of calcium (Figure 2H). Changes in mitochondrial calcium are known to regulate processes such as apoptosis, autophagy, oxidative phosphorylation and crosstalk with the ER [104]. Additionally, the mitochondria serve as a buffering system for cytosolic calcium, where cytosolic calcium is rapidly taken up by the mitochondria. This to prevent excessive calcium accumulation near receptors, which could otherwise disrupt cellular signaling [105,106]. There are various methods to monitor calcium, including dyes that shift wavelength (Fura-2) [107] and indicators of which emitted fluorescence increases upon calcium binding (Fluo-3/Fluo 4) [108]. However, these are not exclusively specific to mitochondrial calcium levels. To circumvent this, cells stained with Fluo-4 can be permeabilized to eliminate the cytosolic Fluo-4 signal, although this limits the imaging duration [109]. A rhodamine-based dye, Rhod-2, can be used to effectively assess mitochondrial calcium. However, even this dye is not perfect, since some of it can accumulate in the liposomes or the cytosol, and the dye can diffuse out of the mitochondria [109]. Rhod-2 has successfully been applied to monitor mitochondrial calcium fluxes in cardiomyocytes and HeLa cells, for example [110,111]. Genetically encoded calcium indicators can also be used, which emit fluorescent signals after binding to calcium. Mitochondrial calcium indicators have been created by generating mitochondria-targeted aequorin and fluorescent probes, enabling mitochondrial-specific calcium imaging [112,113]. A major disadvantage of genetically encoded indicators is the relatively larger size of the indicators, which can alter mitochondrial morphology and behavior [114]. Despite these challenges, mitochondrial calcium imaging has provided significant insights into the calcium dynamics in diseased myocardia and mitochondria–ER crosstalk [115,116]. Calcium dynamics can successfully be imaged with single-photon confocal techniques, like LSCM and SDCM, as well as two-photon microscopy [117]. Given the potential of fast changes in calcium fluxes, SDCM is most suitable because of its high imaging speed.

**Figure 3:**
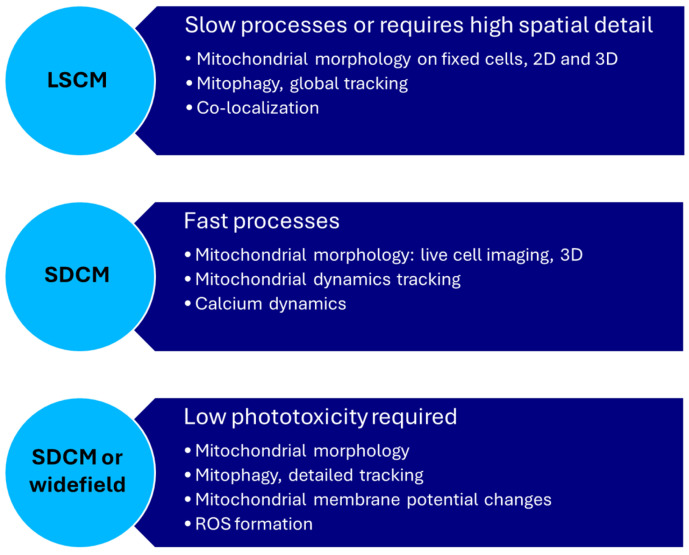
Overview of the three commonly used microscopy techniques and their suitability to various mitochondrial imaging applications. LSCM: laser scanning confocal microscopy; SDCM: spinning disk confocal microscopy.

In summary, the wide variety of applications have allowed mitochondrial imaging at the cell level to become a very common practice. Although specialized microscopy techniques are not necessarily required for any of these applications, each microscopy type offers its unique advantages and disadvantages (Figure 3). In general, LSCM is a widely available and easy-to-use technique which is mostly beneficial in fixed cells. It allows for high-resolution images without problems caused by lower temporal resolutions and phototoxicity (Table 2). Widefield microscopy is similarly widely available, user-friendly, and more suitable for live cell imaging than LSCM. However, when available, the advantages of SDCM in terms of spatial resolution and imaging depth result in distinctly better images than those obtainable with widefield microscopy. While light-sheet microscopy is beneficial when prolonged imaging of the same sample is preferred, the specific advantages of light-sheet microscopy in terms of imaging speed are not required for imaging cells. The same applies to wo-photon microscopy in the imaging of thicker samples. The unique advantages of both microscopy techniques become more important when imaging tissues.

## 4. Imaging Mitochondria in Tissues

The use of imaging at the cell level to study mitochondria (i.e., on cultured cells), as discussed in the previous section, is very common due to the ease of controlling and manipulating the environment in which the cells grow. However, while this approach yields valuable insights, it falls short in capturing the complexity of cellular behavior in vivo. In contrast, imaging studies carried out on tissues better approximate the physiological conditions found in both healthy and diseased states. Mitochondrial studies at the tissue level can be conducted using human samples, animal samples, or organoids. While the advantage of working with human tissue samples is that it leads to results that are directly applicable to human physiology and pathophysiology, it comes with several challenges. Human tissue in a biopsy yields a limited amount of material that often has to be used for multiple assays. Additionally, biopsies cannot always be taken from the organ of interest. In mitochondrial studies, generally, either skin or muscle biopsies are used, since these are relatively easy to obtain. In contrast, biopsies from the kidneys, liver, and brain are not commonly performed, although these organs are also regularly affected by mitochondrial dysfunction. Typically, biopsies are sectioned and only a few slices undergo staining and imaging, which does not provide information on the cellular structures across tissue layers. As a result of the difficulty in obtaining biopsies for some human tissues, many studies opt for animal models or organoids as a model for human tissues. These alternatives not only allow for sectioning and subsequent staining, but also enable 3D imaging to visualize the native tissue structure in more detail. However, animal tissues do not correspond perfectly to human tissues, and while organoids are more complex than single-layer cell cultures, they do not completely mimic human tissues either.

Imaging of tissue sections is generally performed with brightfield microscopy in combination with (immuno)histochemical, (I)HC, staining techniques. One of the first methods for HC mitochondrial staining was modified Gomori trichrome staining, which results in the dark, red staining of fibrous structures called ragged red fibers (RRFs) [118,119]. RRFs are the result of an increase in subsarcolemmal and/or intermyofibrillar mitochondria, mostly in type I muscle fibers. The aggregates of mitochondria cause the muscle fibers to have an irregular shape, leading to their ‘ragged’ appearance [119]. RRFs are indicative of multiple mitochondrial disorders, such as Myoclonic Epilepsy and Ragged Red Fibers (MERRFs), mitochondrial myopathy, encephalopathy, lactic acidosis, stroke-like-episodes (MELAS) and Kearns-Sayre syndrome, and is mainly associated with mtDNA defects [7,120,121,122]. The appearance of RFFs in biopsy samples of people with a suspected mitochondrial disorder can aid in the diagnosis of these patients.

While imaging of RRFs has been clinically useful, mitochondrial accumulations are not the only possible cause of RRF formation [7]. This means that additional staining is necessary to uniquely diagnose a patient. Nowadays, this is mostly performed through the use of enzymatic HC stains of mitochondrial enzymes, thereby ensuring that the staining phenotype uniquely originates from the mitochondria [7]. Succinate dehydrogenase (SDH) and cytochrome c oxidase (COX) enzymatic HC staining is common in mitochondrial research. In muscle tissues with an accumulation of mitochondria resulting in RRF formation, SDH staining reveals a similar pattern to that of RRFs, originating from the accumulated mitochondria within these fibers [7]. Beyond its role in RRF, SDH HC can be used to distinguish type I (oxidative) and type II (glycolytic) muscle fibers, as well as to detect deficiencies in complex II of the mitochondrial respiratory chain. Similarly, COX staining plays a critical role in the phenotypical investigation of mitochondrial disorders. For instance, a mosaic pattern of COX-deficient and COX-positive muscle fibers is indicative of a heteroplasmic mtDNA mutation that affects the expression of mtDNA-encoded genes [7]. Beyond SDH and COX HC staining, antibodies against a wide variety of mitochondrial proteins—such as VDAC, COX7C or Drp1—can be used to visualize the distribution of these proteins within a tissue [123,124], although this approach is more commonly performed with fluorescence imaging. The advantage of using (I)HC on tissues is that it allows for the visualization of dysfunction in the respiratory chain at a cellular level, in contrast to biochemical assays that require the homogenization of the tissue and thus obscure such details. However, the efficacy of staining techniques is dependent on the freshness or preservation state of the tissue. Morphological alterations can arise if the tissue is not adequately fixed and/or sectioned, underscoring the importance of meticulous sample preparation in preserving the integrity of histological analyses [7].

Imaging of (I)HC staining using brightfield microscopy is easy to perform and offers an overview of the cells in the tissue, as well as the general distribution of proteins and complexes within cells in some cases. However, such staining does not allow the visualization of more detailed structures, while brightfield microscopy cannot be used to image tissues in 3D. For these applications, the various types of fluorescent microcopy previously described can be used. As for cellular imaging, live cell imaging of fluorescently stained tissue sections is possible, although this is rarely performed. In contrast to the process followed in the imaging of cells, tissue imaging is usually performed in fixed tissue using immunofluorescent (IF) staining or, in the case of animal or organoid studies, with fluorescently tagged proteins. Similarly to the process followed in (I)HC staining, fluorescent imaging is applied in tissue sections to visualize the distribution of proteins, such as cytochrome C or TOM20, enabling the visualization of RRFs and the mitochondrial network distribution [125,126,127]. LSCM is very suitable for this purpose, since there are no negative effects of laser exposure on the sample, and the resolution allows for the proper visualization of the mitochondrial network. When specific protein distributions are of interest, super-resolution microscopy techniques, like STED, offer enhanced resolution. However, this increased resolution can be disadvantageous when general mitochondrial morphology is analyzed, as it can result in images that show individual dots instead of a continuous structure [125].

Fluorescent microscopy techniques, specifically two-photon microscopy and light-sheet microscopy, are also suitable for the imaging of organoids and tissues in 3D. These microscopy techniques allow for the examination of different tissue layers and interactions between different cell types, neither of which is visible in thin slices or under widefield microscopy. Two-photon microscopy, for instance, has been used to map neurons and vasculature in mouse brain tissue, during which a high resolution and contrast were achievable at a 530 µm depth [128]. Similarly, light-sheet microscopy has been applied to map complete organoids in 3D [129,130]. Despite the advantages of imaging larger tissue sections and the emergence of the mitochondria-specific uses mentioned above, the application of two-photon and light-sheet microscopy for the imaging of mitochondria in tissues is currently very limited.

With fluorescent imaging techniques like two-photon microscopy and light-sheet microscopy, imaging of larger tissues and even complete organs has become feasible. Ex vivo imaging of mitochondria in organs has successfully been applied in the soleus muscle of mice and in drosophila brains [131,132,133]. For this, transgenic animals can be used, or labeling can be performed by incubating the organ with a mitochondria-specific dye or antibody [131,132,133]. Following labeling, the organ of interest can immediately be imaged. As lengthy imaging times are not problematic and the entire organ is accessible, the resulting images have very high spatial detail. Both two-photon imaging and light-sheet microscopy are suitable for achieving the required imaging depth. However, if tissue clearing can be performed, light-sheet microscopy also allows for the imaging of a larger field of view and imaging at higher speed, which can be beneficial when larger organs are imaged.

In summary, mitochondrial imaging at the tissue level predominantly relies on the (I)HC staining of tissue sections combined with brightfield microscopy, and plays a crucial role in patient diagnosis. However, the potential of modern fluorescent imaging techniques to enhance our understanding of mitochondrial behavior in deeper tissue layers and complete organoids remains underutilized. This is likely due to the relatively recent developments in organoid cultures and microscope developments. Currently, only a few studies have applied these microscopy techniques in mitochondrial tissue imaging. Incorporating these advanced imaging techniques could have a significant impact. Examining changes in mitochondrial structure throughout tissue layers is particularly relevant for research into mitochondrial disorders affecting the brain, and could shed light on how brain structures are differentially affected. Moreover, the field of brain organoid technology is rapidly developing, creating opportunities for mitochondrial research [134,135]. For the visualization and tracking of changes in mitochondrial behavior in organoid disease models, light-sheet microscopy is a very suitable technique. This approach is promising for the exploration of the pathophysiology of mitochondrial disorders in a controlled in vitro environment, enabling the evaluation of disease progression, the response to potential treatments, and the effectiveness of gene therapies.

## 5. Imaging Mitochondria in Organs

Gaining insights into the complexity of tissue structures, as well as insights into the effect of mitochondrial (dys)function on surrounding tissues, requires imaging of the tissue or organ in a native state. This can be achieved through ex vivo and in vivo microscopic imaging techniques. However, as microscopic imaging is very invasive, it can only be used in animal models or post mortem human tissue. Alternatively, non-invasive imaging methods, such as positron emission tomography (PET) and magnetic resonance imaging (MRI), can be used for both animal and human applications.

With invasive microscopic techniques, obtaining a physiologically representative view of the organ of interest can be achieved through in vivo fluorescent imaging. labeling is achieved through the injection of a dye, like TMRM, either intravenously or into the retro-orbital sinus in rodents [136,137]. For studies on zebrafish, the dye can also be added to the water for several days [133]. Alternatively, transgenic animals expressing a fluorescent marker can be used [138,139]. Microscopic in vivo imaging is limited to smaller animal models due to its required space, and it requires a specific microscope set-up. Moreover, in most animal models, imaging organs in vivo is not possible without surgical intervention. This is because the available equipment is not capable of imaging through all other tissue layers in front of the organ of interest. Intravital imaging of the liver or kidneys can be performed, for which the animals are put under anesthesia and the organ is exposed before imaging [140], while brain imaging requires the removal of the skull [136]. Recently, a new approach has been developed that allows imaging through bone, thus making skull removal redundant. However, surgical removal of the skin and periosteum remains necessary [141]. Uniquely, zebrafish embryos can be imaged without intervention as they develop outside the uterus and are completely transparent for the first 5–6 days after fertilization [142]. Therefore, zebrafish are a very useful model with which to study embryonic development, particularly in the use of specific transparent zebrafish lines to study further development [143]. Although in vivo imaging is not yet commonly performed in mitochondrial research, it has been used to study the behavior of neuronal mitochondria and calcium uptake in astrocytes and neurons in various animal models [144,145,146]. Additionally, this approach has been used to assess the in vivo distribution of mtDNA mutations [147] and mitochondrial structure during acute kidney injury [137]. Furthermore, protocols have been developed to image mitochondria in kidneys [140], neurons, and bone marrow [141]. Mitochondrial two-photon dyes, such as FO2, Rd1 MitoG2, and MitoY2, have specifically been developed for in vivo application [133,136,148]. Most mitochondrial in vivo imaging is performed with two-photon microscopy since it allows for high-resolution imaging deep into the organ of interest. Light-sheet microscopy can also be very suitable, offering a high resolution and imaging speed. However, light-sheet microscopy’s requirement of tissue clearing before imaging limits its use in living animals, except in transparent models like zebrafish and *C. elegans* [149]. In these animal models, light-sheet microscopy’s decreased phototoxicity and imaging speed are advantageous over two-photon microscopy.

A much less invasive way of imaging is through PET scans. This technique uses radioactive tracers to measure physiological functions like blood flow and metabolism [150]. The radioactive tracers are injected and then distribute within different tissues; their signals are then picked up by detectors in the PET scanner [150]. While the spatial resolution of PET is significantly lower than that of microscopy techniques (~5 mm) [151], its spatial coverage is far more extended (e.g., to image a complete organ), and its limited invasiveness allows for applications in humans.

In mitochondrial research, PET scans can be used in two ways: directly, by using tracers targeting the mitochondria, and indirectly, through correlates of mitochondrial dysfunction, such as oxidative stress. One example of direct tracing is through the use of PK11195. The [11C]PK11195 PET tracer has mainly been used as a marker of neuroinflammation [152,153] even though it has also been suggested to be useful for the detection of microglial accumulation [154]. Additionally, since [11C]PK11195 selectively binds to the TSPO translocator in the outer mitochondrial membrane, it can also be used to study mitochondria [155]. [11C]PK11195 PET imaging of the brain in mitochondrial disease patients showed a correlation between abnormal tracer binding and clinical severity [155]. Beyond the general increase in tracer binding found in neuroinflammation and microglial accumulation, tracer binding varies throughout the brain, suggesting that this tracer may be used as a non-invasive biomarker for the involvement of the central nervous system in mitochondrial diseases [155]. The availability of such a marker could be very advantageous for the tracking of disease progression in studies aimed at designing novel therapies. A different tracer, 4-[18F]fluorobenzyl-triphenylphosphonium ([18F]BnTP), localizes to the mitochondrial inner membrane in a voltage-dependent manner [8]. This tracer was successfully used to study mitochondrial membrane potential and mitochondrial heterogeneity in lung tumors in mice [8]. Additionally, it could be used to detect complex I inhibition, showing the potential of [18F]BnTP as a biomarker to guide complex I inhibitors in cancer treatments [8] and to monitor complex I disorders. However, [18F]BnTP is not currently used in human research, as a number of challenges (e.g., reproducible quantification) need to be addressed before clinical translation can be achieved [156]. A third radiotracer, [18F]BCPP-EF, can also be used to assess mitochondrial complex I activity and has been applied in human studies [157,158]. With the availability of such tracers, there is an opportunity to start assessing mitochondrial functioning with limited invasiveness in cancer and neurodegenerative disorders, for example.

PET radiotracers that do not directly measure mitochondrial activity can also be useful in studying mitochondrial dysfunction. Tetraphenylphosphonium (TPP+) is used to measure membrane potential, and PET tracer [18F]TPP+ was shown to be sensitive to changes in mitochondrial membrane potential [159,160]. Subsequently, researchers showed that this tracer can be used to assess doxorubicin-induced cardiotoxicity in pigs through the monitoring of differences in membrane potential [160]. While the study conditions were not completely representative of the build up of doxorubicin in cancer treatment, it does show potential for the monitoring of cardiac dysfunction as a result of doxorubicin exposure [160]. Similarly, a [62Cu]ATSM PET radiotracer has been applied to determine oxidative stress, which is known to be increased in mitochondrial dysfunction [161]. This tracer has been used to show increased oxidative stress levels in the brains of patients with mitochondrial disease, Parkinson’s disease and amyotrophic lateral sclerosis [161], indicating the a role of mitochondrial dysfunction in the neurodegenerative processes of these disorders.

Another non-invasive imaging technique used in mitochondrial imaging is MRI. MRI uses the natural abundance of protons in the body, from which a signal can be measured when positioned in a strong magnetic field. Variation in this signal over time (i.e., T_1_: horizontal relaxation; T_2_: transverse relaxation) varies with different tissue properties, allowing the reconstruction of images that highlight different tissue properties [162].

While MRI cannot be used to image mitochondria directly, it can be used to study the effect of mitochondrial dysfunction on organs. Currently, MRI is used in the clinic to aid patient diagnosis [3,163,164,165,166,167], and can be applied in muscles or the brain. While MRI of the brain is regularly performed, MRI of muscles for the purpose of diagnosis, for instance in the case of mitochondrial myopathy [167], is rare. Instead, diagnosis is more commonly based on tissue biopsies in combination with genetic testing. When applied in the brain, significant brain abnormalities can be found based on MR data. These include a global delay in myelination and cerebral and cerebellar atrophy [3]. Aside from general changes, different MRI findings point to the presence of specific mitochondrial disorders. For example, patients with Leigh syndrome, a progressive neurological disease, show bilateral gray matter lesions in the brainstem and basal ganglia, as observed in T_2_-weighted images [168,169]. Furthermore, in a significant number of patients with Leigh syndrome, hyperintensities in the thalamus are found, while white matter lesions are less common [3,168]. Using diffusion-weighted imaging (DWI), which provides a measure of structural integrity at a microanatomical level, decreased apparent diffusion coefficient (ADC, quantifying diffusion) values were found for Leigh syndrome patients, especially in the substantia nigra and basal ganglia [169].

In patients with MELAS, a mitochondrial disorder that mainly affects the nervous system and muscles, stroke-like lesions affecting the cortical gray matter can be regularly seen. Differently from Leigh patients, stroke-like lesions in MELAS patients are mostly restricted to the cortical gray matter. While all parts of the cortex can be affected, lesions in the occipital and parietal lobe are twice as common as those in the temporal lobe and four times as common as frontal lobe lesions [170]. Various MR contrasts can be employed to visualize the stroke-like lesions. Using T_2_(-FLAIR) imaging or T_1_-weighted imaging, they show up as hyper- or hypointensities, respectively [165,166]. Arterial spin labeling (ASL) imaging, which provides a measure of blood flow, shows hyperperfusion in stroke-like lesions [165]. DWI can, moreover, differentiate between acute and chronic stroke-like lesions. Specifically, DWI shows hyperintensity with increased ADC in the affected subcortical white matter in acute lesions, while normal diffusivity is found in chronic lesions. This provides critical information in the diagnostic workflow, as it provides an estimate of disease severity [3,165]. That is, while acute lesions are often transient, chronic lesions eventually result in gray matter atrophy in the affected area [165].

Patients with mutations in POLG, the mitochondrial DNA polymerase, also commonly present with stroke-like lesions. These are mostly present in the occipital lobe and can occasionally be found in the parietal and frontal lobe [164,171,172]. Additionally, focal lesions can be found in the cerebral cortex, represented by hyperintensities on T_2_-and T_2_-FLAIR-weighted images, while chronic lesions of the thalamus, cerebellum and inferior olivary nuclei are also found [164,171]. Slowly progressive cerebral atrophy is regularly found as well, which mirrors the severity of the cognitive impairment and often accelerates following exacerbation episodes [164].

Aside from measures of macroanatomy, microanatomy, and blood perfusion, MR can also be used to measure metabolite concentrations through Magnetic Resonance Spectroscopy (MRS). The most commonly used nucleus for MRS is hydrogen (^1^H), since ^1^H is abundant (ensuring a relatively high signal), and ^1^H MRS can be performed with the same hardware as that used for other MR scans; ^1^H-MRS can be used to distinguish various metabolites, including lactate, phosphocreatine, choline and N-acetyl-L-aspartate (NAA), among which changes in lactate and NAA are most commonly observed in mitochondrial disorders [164,172,173,174]. Lactate elevations are observed in lesioned brain areas of patients with POLG mutations, MELAS, and Leigh syndrome, and are indicative of mitochondrial dysfunction. Simultaneously, these patients often present with decreased NAA levels, which is an indication of neuronal compromise or loss [164,172,173,174]. Aside from lactate and NAA, an increase in the choline/creatine ratio can be found in Leigh syndrome patients, while reduced glutamine and choline have been reported in MELAS patients [173,174]. Beyond ^1^H, MRS employing other nuclei such as carbon-13 (^13^C) and phosphorous-31 (^31^P) can provide additional information on metabolite production and degradation [3]. However, while it has been used in muscle [175], the application of ^13^C or ^31^P MRS to mitochondrial disorders is rare, due to scanner hardware requirements and the need for labeled substrates [3].

Due to its non-invasive and versatile nature, MRI has become a useful tool for diagnosing mitochondrial disorders. However, the potential of MRI extends beyond diagnosis, and represents a major opportunity for improving the understanding and tracking of mitochondrial disorders. For example, using MRI to monitor disease progression could help to determine whether acute stages are transient or evolving into chronic disease stages, and to assess the effectiveness of symptomatic treatments (e.g., anti-epileptic drugs). Moreover, MRI could enable research into the correlation between symptoms and specific changes in the brain or disease severity. Gaining a better understanding of these correlations could provide insights into how brain areas differently compensate for energy deficits, and at what point in the disease’s progression compensation is no longer feasible. Knowledge on the differential vulnerability of brain areas could be used as a starting point to further study disease mechanisms in these areas.

**Figure 4:**
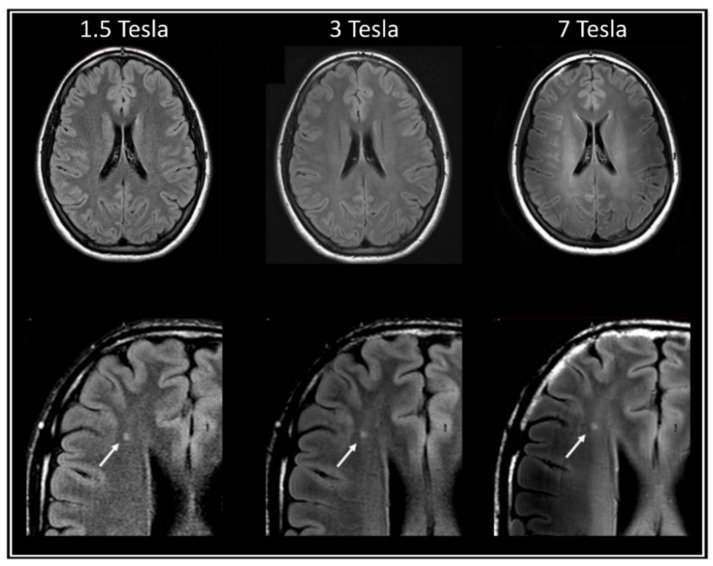
Impact of magnetic field strength and acquisition contrast on MRI images. Comparison of the effects of a 1.5, 3 and 7 Tesla magnetic field strength on image resolution for a T_2_-FLAIR-weighted image. The arrow indicates a white matter lesion (images from Zwanenburg, J.J.M. et al. [177], obtained with permission).

Two relatively recent developments have increased the potential of using MRI in mitochondrial research. First, significant advances in ultra-high-field MRI technology, at 7 Tesla (7T) or beyond, have made these systems more accessible for healthy and clinical populations. Compared to MR at a clinical field strength, which is generally 1.5 or 3 Tesla, 7T MRI profits from a higher signal-to-noise ratio [176], which translates into a higher spatial resolution (Figure 4). This is particularly useful for detecting small changes, such as those expected to be present in early disease stages, the detection of which is crucial for monitoring disease progression.

Second, the use of multiple MR contrasts in a single study, for instance T_1_, T_2_* and DWI, is becoming increasingly common. Previous studies often only looked at a small subset of the possible MR contrasts, precluding a full assessment of the brain phenotype accompanying a mitochondrial disorder [3,163,164,165,166]. However, a recent study taking advantage of the versatility of MRI to study patients with the m.3243A>G mutation observed changes in the brains of patients across contrasts [9,178]. Specifically, the researchers showed consistent changes in cortical gray matter and enlarged ventricles in patients with a high mutation load. These changes were linked to brain areas involved in auditory processing and attentional control, in line with patient symptoms [9]. Additionally, changes in cerebellum gray matter were found, with indications of a reduced concentration of intracortical myelin and iron, as well as alterations in functional cortical connectivity [178]. Not all changes were visible across MR contrasts. For example, T_2_*-weighted images for m.3243A>G patients showed changes across the surface of the occipital cortex that were not found in the other contrasts. This highlights the added value of using multiple MR contrasts, allowing investigators to detect tissue- or function-specific deviations. Moreover, it enables the identification of more severely damaged brain regions, which typically show consistent differences compared to controls across multiple MR contrasts. The further insights into brain pathology that can be obtained by multiple scanning contrasts and ultra-high-field MRI may prove useful in the development of a mechanistic understanding of mitochondrial disorders. When changes are identified earlier, they can give an indication of which brain regions are most vulnerable. This can not only help aid the diagnosis process, but can also be used in combination with different spatial scales to correlate pathological brain changes with underlying molecular alterations. For instance, observations of mitochondrial structure, membrane potential and ROS production in neurons can provide information on the metabolic changes that result from mitochondrial dysfunction, which can be translated into cerebral blood flow levels and can be considered an indication of metabolic activity in the brain.

## 6. Conclusions

**Figure 5:**
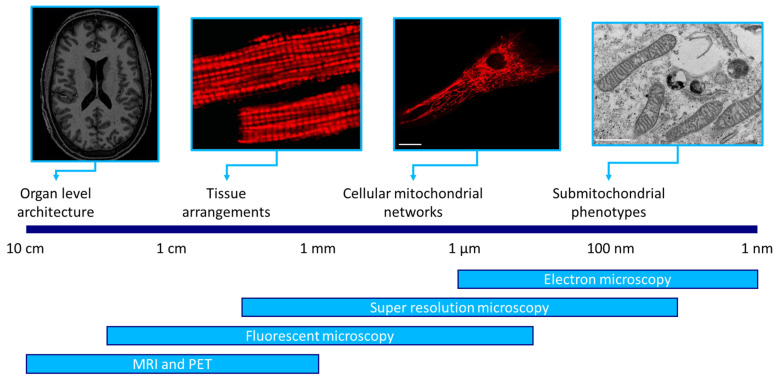
Mitochondrial imaging techniques across scales. All imaging techniques can span various spatial scales; however, a complete picture of all levels can only be obtained by combining various techniques. Tissue image from Kuznetsov, A.V. et al. [179]. Scale bars from left to right indicate 15 µm and 1 µm.

This review of mitochondrial imaging across different spatial scales highlights the versatility of imaging approaches, ranging from the nanoscale resolutions achievable with super-resolution microscopy and electron microscopy to the macroscopic perspectives made possible through imaging techniques such PET and MRI (Figure 5). In addition to pointing out the importance of selecting the appropriate technique for each application, an overview of the versatility in mitochondrial imaging highlights another important point: there is significant opportunity in mitochondrial research.

By integrating data obtained across different spatial scales, researchers can gain a more comprehensive understanding of mitochondrial biology. Currently, the translation from mechanisms underlying mitochondrial function and dysfunction at a cellular level to phenotypical changes in tissues and organs is difficult. While imaging alone cannot provide complete mechanistic insights, the unique possibility of applying imaging on multiple spatial scales can aid in the translation of knowledge between different layers. For instance, post mortem MRI could be used to define regions of interest where the largest differences between healthy and diseased brains occur. These regions can subsequently be removed and processed for smaller-scale imaging techniques to obtain information on mitochondrial structure and protein distribution, for example. This can provide insights into what changes at a cellular level result in the eventual brain phenotype and how this can differ between various brain regions, since the information of the different spatial scales is directly linked.

With continuously advancing technologies and possibilities that allow for an integration of findings across different spatial scales, mitochondrial imaging presents many opportunities to unravel mitochondrial biology in physiological and pathophysiological conditions. Currently, such a multi-scale approach is not very common, likely due to insufficient collaboration between researchers working at different spatial scales. The opportunities highlighted in this review, as well as its support for collaborative efforts, may be crucial to bringing multi-scale mitochondrial research to the next level.

## Figures and Tables

**Table 1:** Characteristics of sub-mitochondrial imaging types.

**Electron Microscopes**	**Spatial Resolution**	**Magnification**	**Sample Thickness**	**Fixed/Live**	**3D Option**
**TEM**	50 pm	50 million	100 nm	Fixed	EM tomography
**SEM**	0.5 nm	2 million	-	Fixed	FIB/SEM
**Fluorescent Microscopes**	**Spatial Resolution**	**Temporal Resolution**	**Imaging Depth**	**Fixed/Live**	**Phototoxicity**
**SIM**	100 nm	10–50 ms	5–15 µm	Both	Low
**STED**	40 nm	1 s	50 µm	Both	High
**STORM**	10–30 nm	>1 s	200 nm	Both	High

**Table 2:** Characteristics of fluorescent microscopy types used for cellular imaging of mitochondria.

Microscopy Type	Temporal Resolution	Imaging Depth	Phototoxicity	Availability
**Widefield microscopy**	5–50 ms	15 µm	Low	Common
**LSCM**	1 s	80 µm	High	Common
**SDCM**	10–50 ms	40–50 µm	Low	Uncommon
**Two-photon microscopy**	1 s	>150 µm	High	Unique
**Light-sheet microscopy**	5–50 ms	>1 mm	Low	Unique

## Data Availability

No new data were created or analyzed in this study.

## Abbreviations

The following abbreviations are used in this manuscript:

EM	Electron microscopy
ROS	Reactive oxygen species
MRI	Magnetic resonance imaging
TEM	Transmission electron microscopy
FIB/SEM	Focused ion beam/scanning electron microscopy
SIM	Structured illumination microscopy
STORM	Stochastic optical reconstruction microscopy
STED	Stimulated emission depletion microscopy
SMLM	Single-molecule localization microscopy technique
SEM	Scanning electron microscopy
LSCM	Laser scanning confocal microscopy
SDCM	Spinning disk confocal microscopy
MMP	Mitochondrial membrane potential
PAGFP	Photoactivatable green fluorescent protein
FRAP	Fluorescence Recovery After Photobleaching
FACS	Fluorescence-activated cell sorting
MCU	Mitochondrial calcium uniporter
ER	Endoplasmic reticulum
(I)HC	(Immuno)histochemical
RRFs	Ragged red fibers
MERRF	Myoclonic Epilepsy and Ragged Red Fibers
MELAS	Mitochondrial myophathy, encephalopathy, lactic acidosis, and stroke-like-episodes
mtDNA	Mitochondrial DNA
SDH	Succinate dehydrogenase
COX	Cytochrome c oxidase
IF	Immunofluorescent
PET	Positron emission tomography
[^18^F]BnTP	4-[^18^F]fluorobenzyl-triphenylphosphonium
TTP^+^	Tetraphenylphosphonium
DWI	Diffusion-weighted imaging
ADC	Apparent diffusion coefficient
ASL	Arterial spin labeling
MRS	Magnetic Resonance Spectroscopy
NAA	N-acetyl-L-aspartate
7T	7 Tesla
